# Cancer-Related NEET Proteins Transfer 2Fe-2S Clusters to Anamorsin, a Protein Required for Cytosolic Iron-Sulfur Cluster Biogenesis

**DOI:** 10.1371/journal.pone.0139699

**Published:** 2015-10-08

**Authors:** Colin H. Lipper, Mark L. Paddock, José N. Onuchic, Ron Mittler, Rachel Nechushtai, Patricia A. Jennings

**Affiliations:** 1 Departments of Chemistry and Biochemistry, University of California San Diego, La Jolla, CA, 92093, United States of America; 2 Center for Theoretical Biological Physics and Department of Physics, Rice University, Houston, TX, 77005, United States of America; 3 Department of Biology, University of North Texas, Denton, TX, 76203, United States of America; 4 The Alexander Silberman Institute of Life Science, Hebrew University of Jerusalem, Edmond J. Safra Campus at Givat Ram, Jerusalem, 91904, Israel; Weizmann Institute of Science, ISRAEL

## Abstract

Iron-sulfur cluster biogenesis is executed by distinct protein assembly systems. Mammals have two systems, the mitochondrial Fe-S cluster assembly system (ISC) and the cytosolic assembly system (CIA), that are connected by an unknown mechanism. The human members of the NEET family of 2Fe-2S proteins, nutrient-deprivation autophagy factor-1 (NAF-1) and mitoNEET (mNT), are located at the interface between the mitochondria and the cytosol. These proteins have been implicated in cancer cell proliferation, and they can transfer their 2Fe-2S clusters to a standard apo-acceptor protein. Here we report the first physiological 2Fe-2S cluster acceptor for both NEET proteins as human Anamorsin (also known as cytokine induced apoptosis inhibitor-1; CIAPIN-1). Anamorsin is an electron transfer protein containing two iron-sulfur cluster-binding sites that is required for cytosolic Fe-S cluster assembly. We show, using UV-Vis spectroscopy, that both NAF-1 and mNT can transfer their 2Fe-2S clusters to apo-Anamorsin with second order rate constants similar to those of other known human 2Fe-2S transfer proteins. A direct protein-protein interaction of the NEET proteins with apo-Anamorsin was detected using biolayer interferometry. Furthermore, electrospray mass spectrometry of holo-Anamorsin prepared by cluster transfer shows that it receives both of its 2Fe-2S clusters from the NEETs. We propose that mNT and NAF-1 can provide parallel routes connecting the mitochondrial ISC system and the CIA. 2Fe-2S clusters assembled in the mitochondria are received by NEET proteins and when needed transferred to Anamorsin, activating the CIA.

## Introduction

Iron-sulfur (Fe-S) clusters are ancient cofactors found in proteins from all kingdoms of life. Proteins containing iron sulfur clusters perform numerous critical functions including transfer of electrons in oxidative metabolism, photosynthesis, nucleotide metabolism, synthesis of protein cofactors, and are crucial for DNA replication and DNA repair [1, 2]. Biosynthesis of Fe-S clusters occurs by complex protein assembly systems which include scaffold proteins, chaperones, electron transfer proteins and cysteine desulfurases [3]. In mammals there are two distinct sets of Fe-S cluster assembly machinery: the mitochondrial iron sulfur cluster assembly (ISC) system and the cytosolic iron sulfur cluster assembly (CIA) system. The CIA supplies Fe-S clusters for cytosolic and nuclear proteins, including those involved in DNA replication and repair [4]. The mitochondrial ISC system has been reported to be necessary for the activation of the cytosolic system [5, 6]. However the exact nature of the link between the two systems remains unknown. There are several human diseases associated with defects in Fe-S biogenesis, including Friedreich’s Ataxia, sideroblastic anemia, multiple mitochondrial dysfunctions syndrome, and cancer [3, 7].

The human NEET proteins are a recently discovered new class of 2Fe-2S proteins. They are the only known iron-sulfur proteins localized at or near the outer mitochondrial membrane that are at the interface between the mitochondria and cytosol. The best characterized of these are mitoNEET (mNT, CISD1) and nutrient-deprivation autophagy factor-1 (NAF-1, miner1, ERIS, CISD2) (reviewed in [8]). mNT is located on the outer-mitochondrial membrane (OMM) and NAF-1 on the endoplasmic reticulum (ER) and the mitochondrial-associated membrane (MAM) of the ER [9–11]. NAF-1 in the MAM is intimately connected to the OMM via its complex with IP_3_R, which is directly tethered to the OMM pore protein VDAC by grp75 [9, 12]. These NEET proteins are involved in several human diseases including cancer, diabetes, cystic fibrosis, Wolfram syndrome 2, neurodegeneration and muscle atrophy [8, 13–18]. Crystal structures show that both mNT and NAF-1 are homodimers with each protomer coordinating a 2Fe-2S cluster with an unusual 3Cys:1His coordination [19, 20]. The unusual Fe-S cluster coordination by three Cys ligands and one non-Cys ligand has been found in Fe-S cluster transfer proteins [21]. We demonstrated the ability of NEET proteins to transfer their 2Fe-2S clusters to apo-ferredoxin, the gold-standard protein used for Fe-S cluster transfer experiments [22, 23].

The subcellular localization of the NEET proteins at the interface of the cytosol and the mitochondria, as well as their cluster transfer function, led us to investigate whether they connect the mitochondrial ISC system to the CIA system. The cytosolic 2Fe-2S protein Anamorsin (also known as cytokine induced apoptosis inhibitor-1; CIAPIN-1) is an electron transfer protein that is required for an early step of Fe-S cluster assembly by the CIA system [24–26]. In this study we identify Anamorsin as the first known direct physiological 2Fe-2S cluster acceptor for both mNT and NAF-1. Similar to our previous findings with ferredoxin, transfer only occurs when the cluster is in the oxidized [2Fe-2S]^2+^ state and is inhibited by mutation of the His ligand to Cys. The rate of cluster transfer to Anamorsin from mNT or NAF-1 is comparable to those by other known human 2Fe-2S cluster transfer proteins. We show, using biolayer interferometry, that both NEETs form a direct protein-protein interaction with Anamorsin. Furthermore, we report that NEET proteins directly transfer 2Fe-2S clusters to both Anamorsin cluster-binding sites allowing for complete reconstitution of holo-Anamorsin. Because holo-Anamorsin is required for the biosynthesis of Fe-S clusters by the CIA system, 2Fe-2S cluster transfer to apo-Anamorsin suggests that the NEET proteins have an essential role in regulation/activation of cluster assembly by the CIA system.

## Experimental Procedures

### Expression and purification of proteins

The soluble domains of NAF-1 (residues 57–135) and mNT (residues 33–108) were expressed and purified as described previously [19, 23]. The purified NEET proteins contain ≥ 98% cluster occupancy. The human Anamorsin cDNA encoding plasmid (Abgent) was amplified by PCR and subcloned into a pET28-a(+) vector (Novagen) between NdeI and XhoI restriction sites. The vector adds a 6x his-tag and a thrombin cleavage site to the N-terminus of the gene. BL-21 Codon Plus (DE3) RIL cells (Stratagene) were transformed with the pET28-a(+)-Anamorsin construct. Cultures were grown in LB media containing as described previously [27]. 750 μM FeCl_3_ was added to the culture 30 minutes before induction with IPTG. Cells were pelleted by centrifugation and resuspended in 20 mM Tris-HCl pH 8.0, 250 mM NaCl, 5 mM imidazole. Resuspended cells were lysed using an EmulsiFlex-C5 homogenizer (Avestin) and centrifuged. Supernatant containing his-tagged Anamorsin was bound to Ni-NTA agarose (Qiagen) resin, washed with the resuspension buffer and eluted with the same buffer with 300 mM imidazole. Eluted Anamorsin was diluted in 25 mM Tris-HCl pH 7.1 containing 5 mM DTT and further purified using a HiTrap Q HP 5 ml anion-exchange column (GE Healthcare). The protein was eluted by a 150–500 mM NaCl gradient in 25 mM Tris-HCl pH 7.1 and 5 mM DTT. For apo-Anamorsin, the 2Fe-2S cluster was removed by the method described by Kennedy and Beinert [28]. The single cluster site mutants of Anamorsin (C1 and C2) were designed by replacing each of the 4Cys cluster ligands with Ser by site-directed mutagenesis.

### UV-Vis absorption spectroscopy transfer kinetics

Absorption spectra were recorded from 300–800 nm on a Cary 50 spectrophotometer (Varian) with a temperature control unit set at 37°C using a 1 cm pathlength cuvette. Spectra were obtained under aerobic conditions unless otherwise stated. The ratio of absorbance at 423 nm (characteristic of the 2Fe-2S cluster(s) of Anamorsin) to 458 nm (NEET 2Fe-2S cluster signature peak) was monitored as cluster transfer progression. The extent of the cluster transfer reaction is determined and plotted described previously [23] using the equation: Transfer Progress(%) = (R_obs_−R_initial_)/(R_final_−R_initial_)x100%, where R_initial_ is the 423/458 nm absorbance ratio at time 0, R_obs_ is the 423/458 nm ratio at a given time, and R_final_ is the is the 423/458 nm ratio for complete transfer. R_initial_ for transfer from NAF-1 and mNT is 0.78. R_initial_ values for transfer from NAF-1 H114C mutant and mNT H87C mutant are 0.93 and 0.90 respectively. The 423/458 nm absorbance ratio of purified holo-Anamorsin, C1 mutant and C2 mutant (1.04, 1.03, 0.99 respectively) were used as the values for complete transfer (R_final_). For transfer reactions, apo-Anamorsin was pre-incubated with 2.5 mM DTT for 60 minutes to ensure the reduction of its cysteine thiol groups. Kinetic measurements were performed using equimolar concentrations of NAF-1 or mNT to apo-Anamorsin (one NEET with two 2Fe-2S cluster per Anamorsin) in 50 mM Bis-Tris pH 7.0, 100 mM NaCl, 2.5 mM DTT unless stated otherwise stated. The apo-Anamorsin and DTT were preincubated for 60 min prior to the addition of NEET protein.

### Biolayer interferometry

Kinetics of the apo-Anamorsin interaction with NAF-1 or mNT was measured on an Octet Red96 instrument (ForteBio). Apo-Anamorsin was biotinylated by incubation for 30 minutes with EZ-Link Sulfo-NHS-LC-biotin (Thermo Scientific) at a 2:1 molar ratio of biotinylation reagent to protein then buffer exchanged using a PD10 desalting column (GE Healthcare) into 50 mM bis-tris, 100 mM NaCl, 0.1% Tween 20, 0.5 mg/ml BSA, 5 mM DTT, pH 7.0. 3.5 μM biotinylated Anamorsin was immobilized to streptavidin biosensors (ForteBio). The loaded biosensors were equilibrated in the same buffer without DTT. Association was measured over 900 seconds. The association is determined by wavelength shift (nm). Following association, dissociation was determined by transferring the biosensor tip to buffer without NEET protein for a period of 1800 seconds. Controls for non-specific binding for each NEET concentration were run with no loading to the biosensor and subtracted from the corresponding binding data. Data was fit to a 1:1 model for binding to apo-Anamorsin and a 2:1 heterogeneous ligand model for binding to holo-Anamorsin using the Octet software. The data was plotted using Kaleidagraph (Synergy Software).

### Preparation of post-cluster transfer Anamorsin for mass spectrometry and biolayer interferometry

25 μM apo-Anamorsin in 50 mM Bis-Tris pH 7.0 and 100 mM NaCl was pre-incubated with 2.5 mM DTT for 60 minutes. 27.5 μM NAF-1 was then added and the reaction mixture was incubated in an orbital shaker at 37°C for 3.5 hours. After the reaction holo-Anamorsin was purified from NAF-1 using a HiTrap Q HP 1 ml anion-exchange column. The protein was eluted by a 150–500 mM NaCl gradient in 25 mM Tris-HCl pH 7.5. Samples for mass spectrometry were buffer exchanged into 10 mM Ammonium acetate pH 7.5 buffer using Quick Spin Protein columns (Roche).

### Electrospray ionization mass spectrometry

Anamorsin samples in 10 mM Ammonium acetate pH 7.5 with a concentration of ~1 mg/ml were diluted 100 fold in 50% methanol with 0.5% acetic acid and injected into a Quattro Ultima Triple Quadrupole system (Waters). Spectra were acquired in positive ion mode with an m/z range of 500–2000. Mass deconvolution was performed using the MassEnt1 tool in the MassLynx software package.

## Results

### NAF-1 or mNT can transfer their oxidized 2Fe-2S clusters to apo-Anamorsin

**Fig 1:**
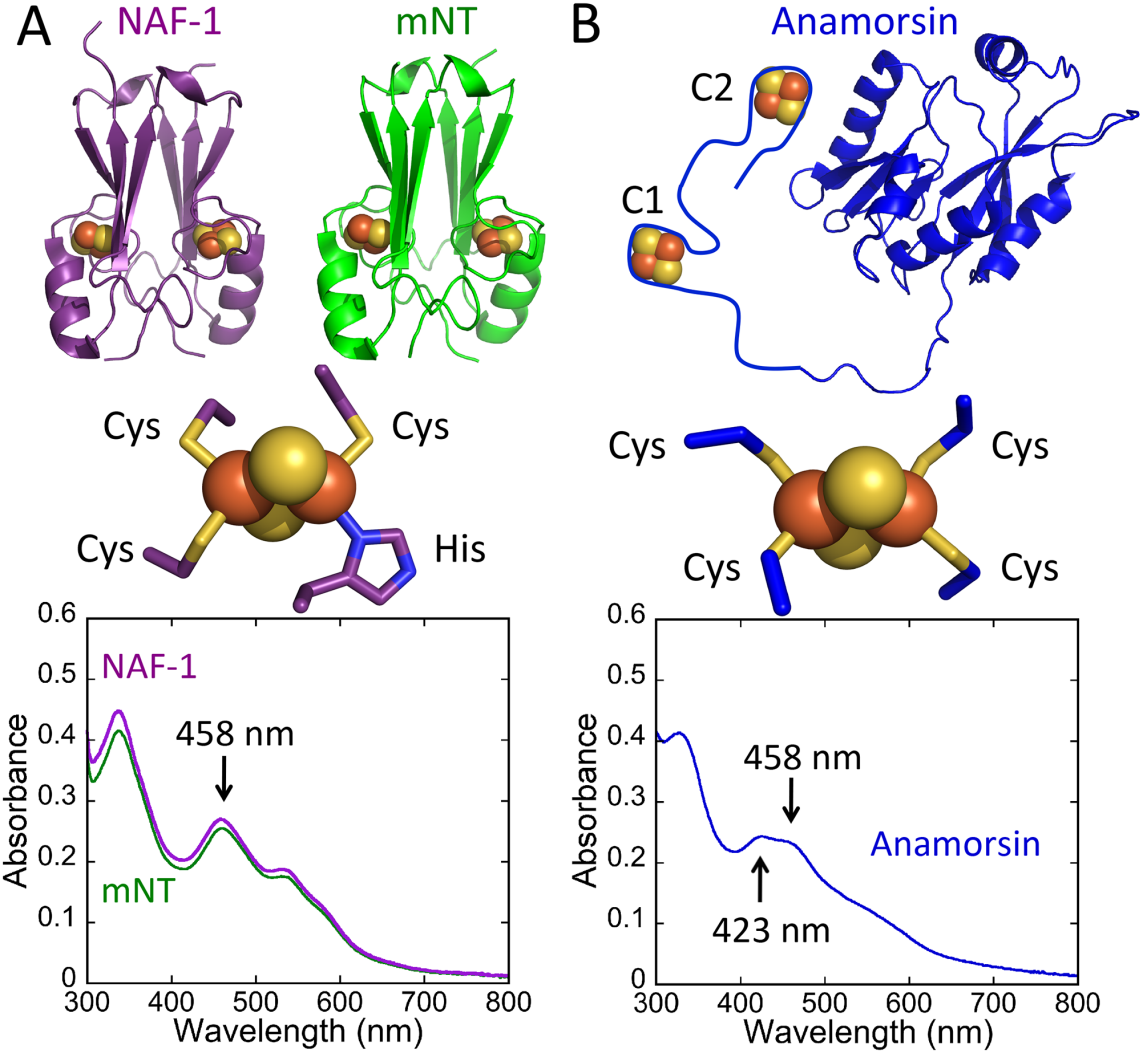
Structures and UV-Vis absorption spectra of NEET proteins and Anamorsin. A. (Top) Crystal structures of mNT (PDB code: 2QH7,), NAF-1 (PDB code: 3FNV); (Middle) NEET 2Fe-2S cluster with 3-Cys:1His coordination; (Bottom) Absorption spectra of 25 μM mNT and NAF-1. B. (Top) Crystal structure of the N-terminal domain of Anamorsin (PDB code: 2YUI) with an added schematic of the unstructured 2Fe-2S cluster binding domain; (Middle) Representative Anamorsin 2Fe-2S cluster with 4-Cys coordination (from ferredoxin, PDB code: 1RFK); (Bottom) Absorption spectrum of 43 μM Anamorsin isolated from *E*. *coli*.

**Fig 2:**
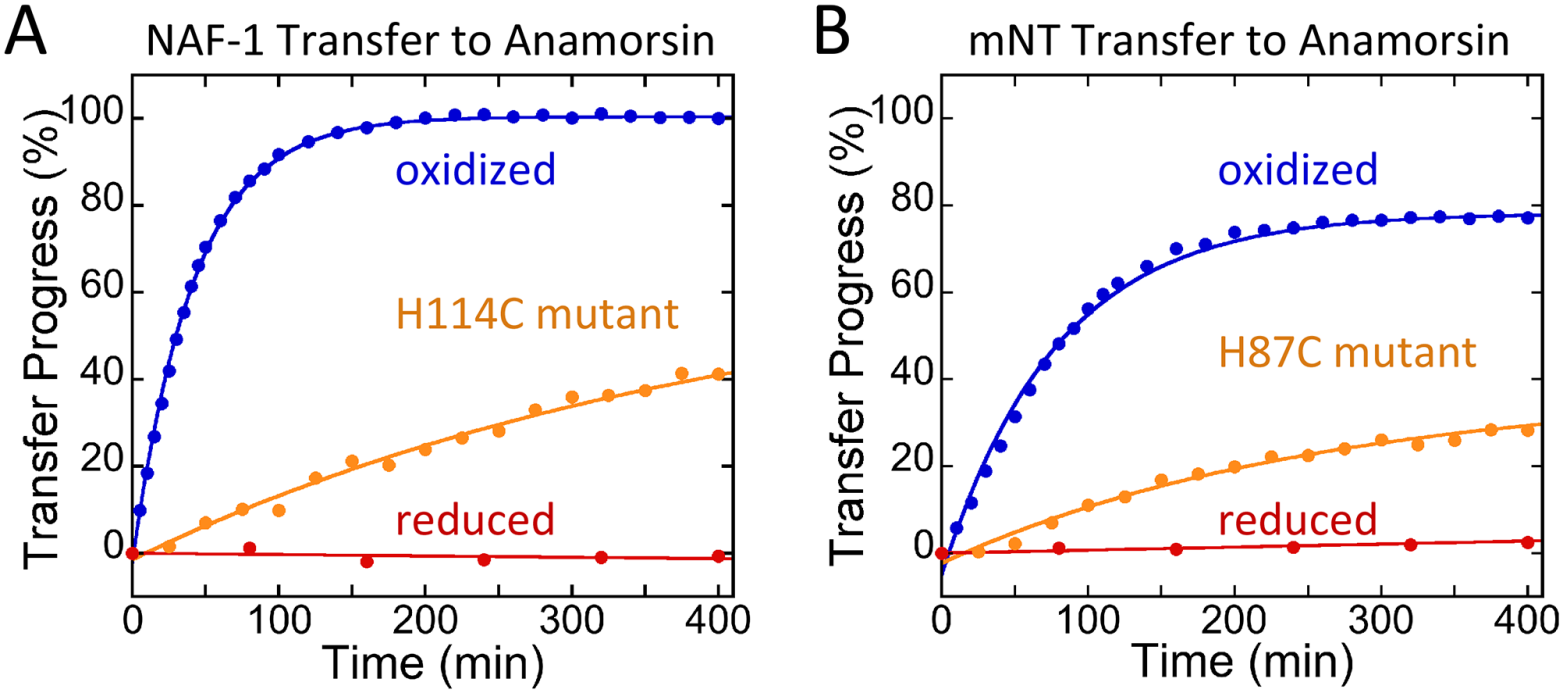
NEET proteins transfer their 2Fe-2S cluster to apo-Anamorsin. The 2F-2S cluster transfer reaction was monitored by UV-Vis absorption spectroscopy. The progress of the transfer was plotted versus time. Cluster transfer from NAF-1 (A) and mNT (B) to apo-Anamorsin occurs only when the 2Fe-2S cluster is oxidized and not when reduced with 10 mM sodium dithionite. Replacement of the coordinating His residue with Cys (H114C for NAF-1 and H87C for mNT) inhibits but does not abolish transfer. All traces shown were obtained with 25 μM NAF-1 or mNT (50 μM 2Fe-2S clusters) and 50 μM apo-Anamorsin in 50 mM bis tris, 100 mM NaCl, 2.5 mM DTT, pH 7.0 at 37°C.

Anamorsin has two 2Fe-2S binding sites, each with a ferredoxin-like 4-Cys cluster ligation that differs from that of the NEETs (3Cys:1His). This difference in ligation gives it a UV-Vis absorbance spectrum that is distinct from that of the NEET proteins, predominantly in the 400–500 nm region. In this region Anamorsin has absorption peaks at 423 and 458 nm, whereas both NAF-1 and mNT have only a peak at 458 nm (Fig 1). We determined the molar extinction coefficient of the Anamorsin 2Fe-2S cluster peak at 458 nm to be 5800 M^-1^cm^-1^ per cluster, while that of the NEETs is 5000 M^-1^cm^-1^ per cluster [29]. We use the ratio of 423 nm to 458 nm to monitor transfer of 2Fe-2S clusters from mNT or NAF-1 to apo-Anamorsin. As transfer occurs we expect the 423 nm peak to rise, and thus an increase in the 423/458 ratio. In contrast loss of the cluster to solution would result in complete loss of visible absorbance in the 400–800 nm region [19]. To prepare the protein for 2Fe-2S cluster transfer studies, the free cysteines of apo-Anamorsin are reduced by pre-incubation with DTT for 60 minutes to ensure that they are ready for cluster ligation. The first scan upon the initiation of transfer shows an oxidized NEET spectrum, which indicates that most of the DTT is oxidized. Reduced DTT will reduce the NEET clusters [30], which inhibits transfer [23]. Subsequently, NAF-1 or mNT was added to pre-reduced apo-Anamorsin and the cluster transfer reaction was monitored by UV-Vis spectrophotometry. The stoichiometry of NAF-1 or mNT to apo-Anamorsin was chosen to provide one 2Fe-2S cluster per Anamorsin as it was previously reported that both cluster binding sites could not be simultaneously reconstituted [24, 27]. Under oxidizing conditions transfer proceeds from both NAF-1 and mNT with no loss of clusters to solution and the data are well fit to a single exponential phase (Fig 2). NAF-1 transfer proceeded to completion whereas mNT transferred ~80% of its clusters showing efficient transfer from either NEET protein to Anamorsin. Scans at select time points can be seen in S1 Fig. Reduced DTT has been shown in some cases to mediate cluster transfer [31]. To test whether this was the case for NEET-Anamorsin, DTT was removed from apo-Anamorsin following 60 min incubation with this agent. The DTT free apo-Anamorsin is readily able to receive the NEET clusters (S2 Fig). This indicates that NEET-Anamorsin cluster transfer is a solely protein-mediated event.

We further tested whether the 2Fe-2S cluster transfer from the NEET proteins to apo-Anamorsin was dependent on the oxidation state of the cluster as we found in previous studies with cluster transfer to apo-ferredoxin [22, 23]. The reduced NEET spectrum has two distinct peaks (S3 Fig), unlike the featureless spectrum of reduced Anamorsin [27]. When the NEET 2Fe-2S cluster is pre-reduced with sodium dithionite no transfer to apo-Anamorsin was observed (Fig 2). This finding shows that NEET cluster transfer to Anamorsin requires oxidized 2Fe-2S clusters as we previously found for transfer from NEET proteins to apo-ferredoxin, as well as observed for transfer from the iron-sulfur assembly protein ISCU to apo-Fd [32].

We additionally tested the importance of the single His ligand of mNT and NAF-1 for efficient cluster transfer. Replacement of the coordinating His residues with Cys in NAF-1 (H114C) and in mNT (H87C) inhibited transfer to apo-ferredoxin [22, 23, 33, 34]. The spectra of NAF-1 H114C mutant and mNT H87C mutant can be seen in S4 Fig. Similarly we find that cluster transfer to apo-Anamorsin was also inhibited by more than 10-fold in these mutants (Fig 2).

### Transfer rates of NEET proteins are similar to those of known human cluster-transfer proteins

**Fig 3:**
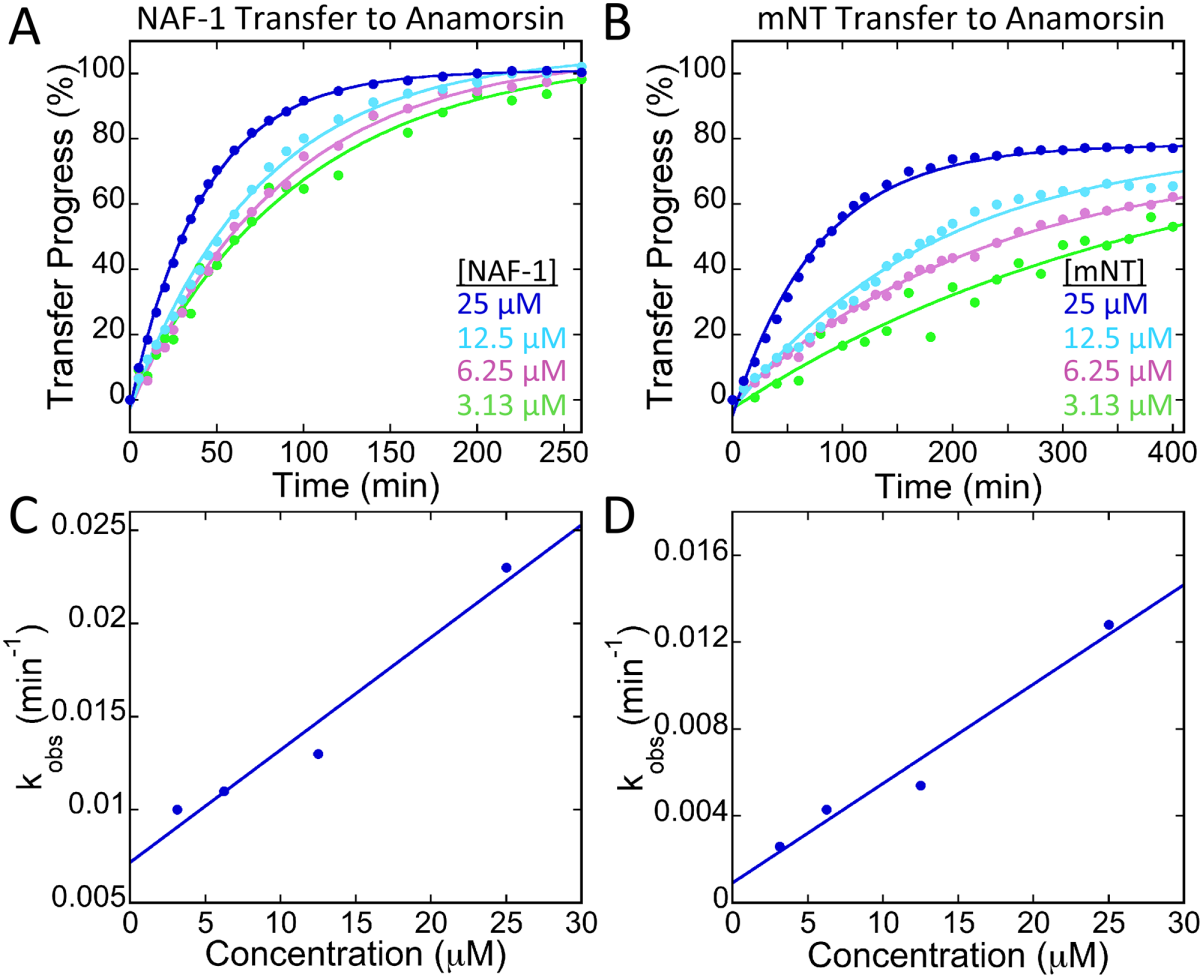
NEET transfer of 2Fe-2S clusters to apo-Anamorsin is second order. NAF-1 (A) and mNT (B) transfer to apo-Anamorsin was monitored by UV-Vis absorption spectroscopy for a series of NEET concentrations. For each NEET concentration the ratio 1 NEET dimer per 2 apo-anamorisn was maintained. The rate constant, k_obs_, is determined from the fit of the data to an exponential rise and is plotted versus concentration for NAF-1 (C) or mNT (D). The slope of the best line fit was used to determine apparent second order rate constants (*k*_2_) for NAF-1 and mNT, which are 600 ± 90 M^-1^ min^-1^ and 460 ± 60 M^-1^ min^-1^ respectively.

Transfer of 2Fe-2S clusters to apo-acceptor proteins has been described as being second order [32, 35]. We therefore tested cluster transfer from mNT or NAF-1 to apo-Anamorsin at different concentrations of the NEET dimer donors (3.13 μM, 6.25 μM, 12.5 μM and 25 μM). The observed rate for each concentration (k_obs_) was linearly dependent on the NEET protein concentration (Fig 3). The apparent second order rate constant (*k*_2_) for each was determined from the linear fits to the k_obs_ values. The *k*_2_ values calculated for NAF-1 and mNT are 600 ± 90 M^-1^ min^-1^ and 460 ± 60 M^-1^ min^-1^ respectively, which are similar to rate constants measured for the human 2Fe-2S cluster transfer proteins ISCA and ISCU [32, 35] (Table 1). Transfer rates of some 2Fe-2S transfer proteins are enhanced by ATP-dependant chaperones. IscU from *Azotobacter Vinelandii* in the presence of the HscA/HscB cochaperone system and necessary cofactors transfers with a reported rate constant that is only slightly greater than those of the NEET proteins [36] (Table 1). An additional similarity between the NEETs and ISCU is the requirement for oxidation of the 2Fe-2S cluster for transfer [32]. Taken together, the NEETs transfer as effectively as ISCU and are likely to function in a similar manner.

**Table 1:** Comparision of NEET 2Fe-2S cluster transfer rates to apo-Anamorsin with other known cluster transfer proteins.

	*k*_2_ (M^-1^ min^-1^)
NAF-1 to apo-Anamorsin	600 ± 90
mNT to apo-Anamorsin	460 ± 60
hsISCA to apo-hsFerredoxin	170 ± 8 [35]
hsISCU to apo-hsFerredoxin	540 [32]
*avIscU to apo-avFerredoxin	800 [36]

*Transfer in the presence of chaperones

Here we demonstrate that 2Fe-2S clusters are transferred to Anamorsin from the NEET proteins. To address the possibility that the NEETs are simply suppliers of iron and sulfide via a release and capture mechanism, we performed transfer in the presence of EDTA (an iron chelator which sequesters free iron and thus inhibits cluster assembly). While free iron and sulfide can spontaneously form clusters on Anamorsin [24, 27], EDTA abolishes the assembly. However, transfer from each of the NEETs to apo-Anamorsin proceeds efficiently in the presence of EDTA (S5 Fig). Therefore, the NEETs’ role in this process is beyond simply supplying iron and sulfide and direct interaction is necessary for efficient transfer.

### NAF-1 or mNT bind directly to apo-Anamorsin

**Fig 4:**
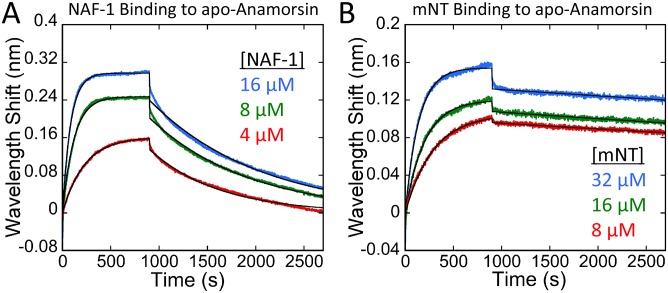
Biolayer interferiometry shows a direct protein-protein interaction of NAF-1 and mNT with apo-Anamorsin. Sensorgrams for the binding of holo-NAF-1 (A) and holo-mNT (B) to biotinylated apo-Anamorsin immobilized to streptavidin-coated biosensors are shown. The association was followed for 900 seconds (rising signal) followed by 1800 seconds of dissociation (decaying signal). The data was fit to a one-to-one model (black curves). On- and off-rates were determined from the fits for each NEET-Anamorsin concentration (shown in Table 2).

To investigate the potential for protein-protein interaction between NEET proteins and Anamorsin we employed biolayer interferometry (BLI). Both NAF-1 and mNT bound directly to immobilized apo-Anamorsin. The association and dissociation curves are shown in Fig 4. On- (*k*_on_) and off-rates (*k*_off_) measured for each interaction are shown in Table 2. The on-rates for NAF-1 and mNT differ by a factor of two, whereas the off-rate of NAF-1 is ~18 fold faster than that of mNT. We also analyzed the binding of the holo-NEETs to holo-Anamorsin (S6 Fig).

**Table 2:** NEET-apo-Anamorsin association and dissociation rates determined by biolayer interferometry.

	*k*_on_ (M^-1^ min^-1^)	*k*_off_ (min^-1^)
NAF-1	(4.7 ± 0.8) x 10^4^	(6.6 ± 0.2) x 10^−2^
mNT	(1.9 ± 0.6) x 10^4^	(3.7 ± 0.5) x 10^−3^

### NEET proteins can transfer to each of the Anamorsin cluster-binding sites

**Fig 5:**
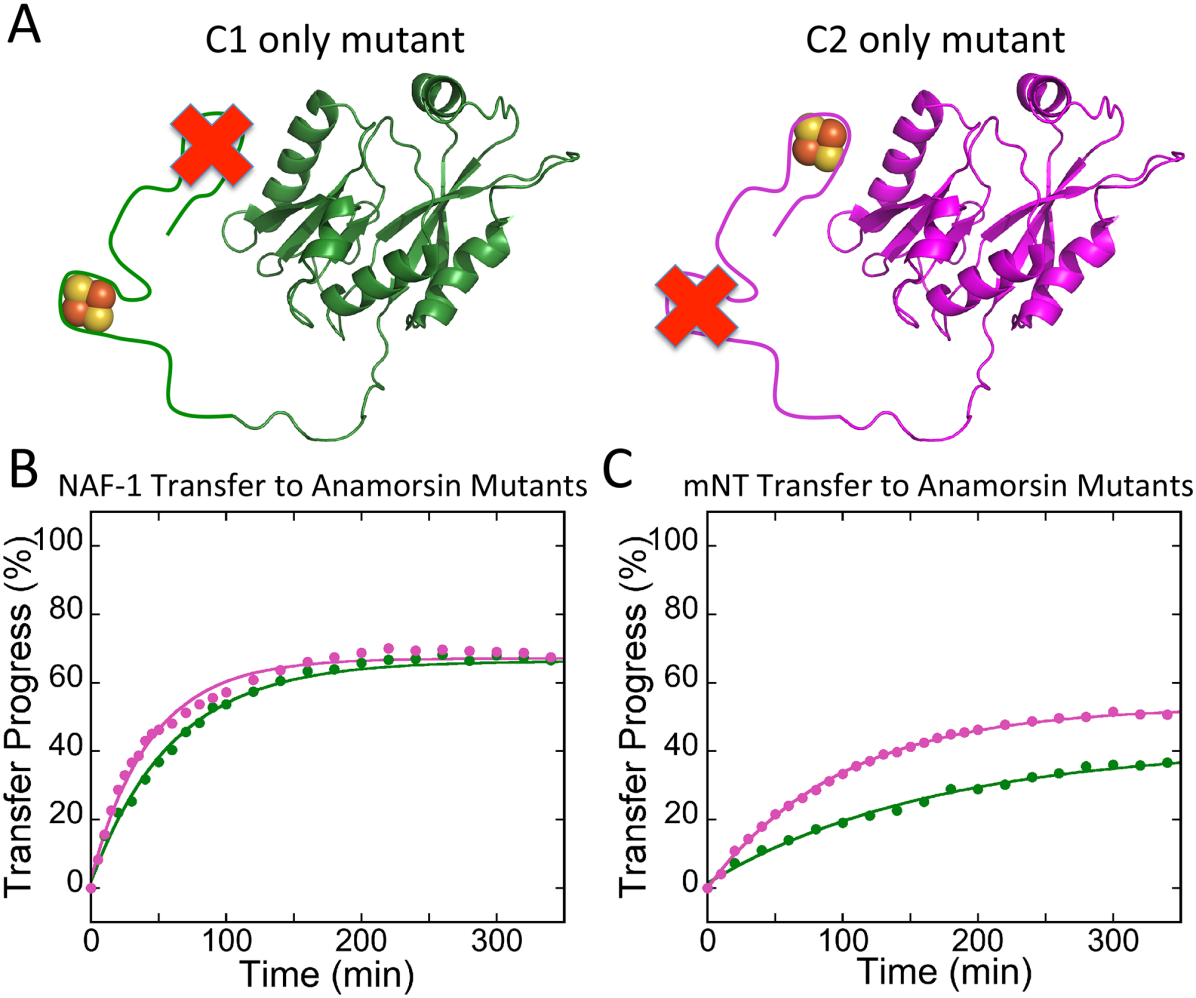
NEET proteins can transfer their clusters to either of Anamorsin 2Fe-2S cluster-binding sites. Anamorsin mutants containing a single cluster-binding site are shown; the other cluster site was disrupted by replacing all of the Cys residues with Ser. (A) Schematics of Anamorsin-C1 (green) and Anamorsin-C2 (magenta) are shown. 2Fe-2S cluster transfer from NAF-1 (B) or mNT (C) to each single cluster binding apo-Anamorsin mutant was monitored by UV-Vis absorption spectroscopy. Traces were obtained with 25 μM NAF-1 or mNT (50 μM 2Fe-2S clusters) and 50 μM apo-Anamorsin.

Anamorsin has two 2Fe-2S cluster binding sites and *in vitro* chemical reconstitution showed that each cluster site could be reconstituted but cluster binding was mutually exclusive [27]. We refer to the first site as C1 and the second as C2 (Fig 5A). Anamorsin mutants containing only a single cluster-binding site were produced in which all four cysteine residues from the other site were replaced with serines to test for possible preferential transfer from mNT or NAF-1 to apo-Anamorsin. The absorbance spectrum for each mutant can be seen in S7 Fig. As shown in Fig 5, each Anamorsin mutant received clusters from each NEET donor protein showing little preference for transfer to either the C1 or C2 acceptor sites.

### NAF-1 homodimers can supply both 2Fe-2S clusters to Anamorsin

**Fig 6:**
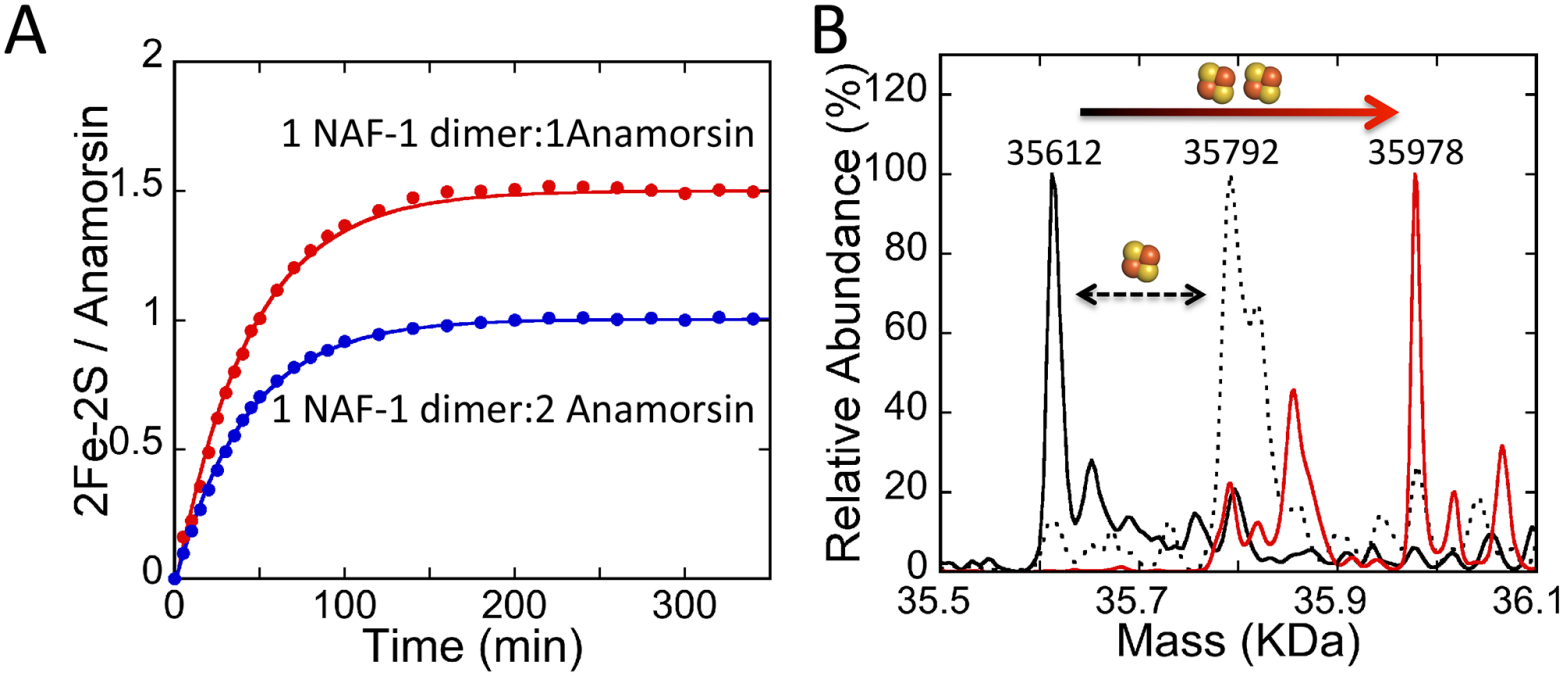
NEET homodimers can transfer their two 2Fe-2S clusters to Anamorsin. (A) 2Fe-2S transfer from 50 μM dimeric NAF-1 to 50 μM apo-Anamorsin (two 2Fe-2S clusters per Anamorsin) (shown in red) is compared to 25 μM NAF-1 to 50 μM apo-Anamorsin (one 2Fe-2S cluster per Anamorsin) (shown in blue). Transfer is near complete for the one 2Fe-2S cluster per Anamorsin sample and is approximately 75% complete for the two 2Fe-2S cluster per Anamorsin sample. The latter result shows that at least half of the Anamorsin is capable of receiving two 2Fe-2S clusters from a single NAF-1 homodimer. (B) Anamorsin following a transfer reaction with NAF-1 was purified and analyzed by ESI-MS (shown in red). Mass spectra of apo-Anamorsin (solid black line) is compared to the major peak for post-transfer Anamorsin (solid red line) which shows an increase in the mass corresponding to the incorporation of two 2Fe-2S clusters. There is also a minor peak at 35856 Da that likely corresponds to Anamorsin with a single 2Fe-2S cluster and an iron ion bound that may be a remnant of a cluster that degraded during the sample preparation process. *E*. *coli*-purified Anamorsin is shown for comparison (dotted line) which shows Anamorsin containing a single 2Fe-2S cluster.

Given the above results, we decided to test whether either a single NEET homodimer could supply 2Fe-2S clusters to both C1 and C2 acceptor sites of a single apo-Anamorsin. Cluster transfer was measured for NAF-1 with stoichiometric donor cluster to acceptor sites (50 μM NAF-1 dimer and 50 μM apo-Anamorsin) (Fig 6A). We focused on NAF-1 because only NAF-1 showed complete transfer with excess apo-Anamorsin (Fig 2). Transfer of ~1.5 2Fe-2S clusters per Anamorsin was found, surprisingly showing that at least half of the Anamorsin proteins accepted two clusters after incubation with NAF-1. We observed no loss of cluster to solution (spectral amplitude). As the data are well fit to a single exponential phase, both clusters are transferred in kinetically indistinguishable steps. The simplest explanation is that both clusters are transferred in a single binding event, although they may be at slightly different rates because of the different amino acid composition around the two Anamorsin cluster-binding sites. The incomplete transfer may be due to formation of intramolecular disulfide bonds in apo-Anamorsin or possibly due to modified cysteine side-chains in apo-Anamorsin. The former was suggested previously for incomplete transfer from ISCU to apo-ferredoxin [32]. To confirm the formation of fully occupied holo-Anamorsin following transfer from NAF-1 we used electrospray ionization mass spectrometry (ESI-MS). The holo-Anamorsin formed by a transfer reaction with NAF-1 was purified from NAF-1 using anion exchange chromatography. The resulting holo-Anamorsin was analyzed by ESI-MS. Additionally we acquired ESI-MS spectra for apo-Anamorsin and Anamorsin as it was purified from *E*. *coli*. The resulting spectra are shown in Fig 6B. The expected mass for the apo-Anamorsin construct is 35614 Da and the expected mass of a 2Fe-2S cluster is 176 Da. We find that the Anamorsin purified from *E*. *coli* is predominantly contains a single cluster, while the major fraction of post-transfer Anamorsin contains two 2Fe-2S clusters; previously chemical reconstitution reported cluster occupancy to be mutually exclusive [27]. This result shows the necessity of direct transfer to activate Anamorsin.

## Discussion

**Fig 7:**
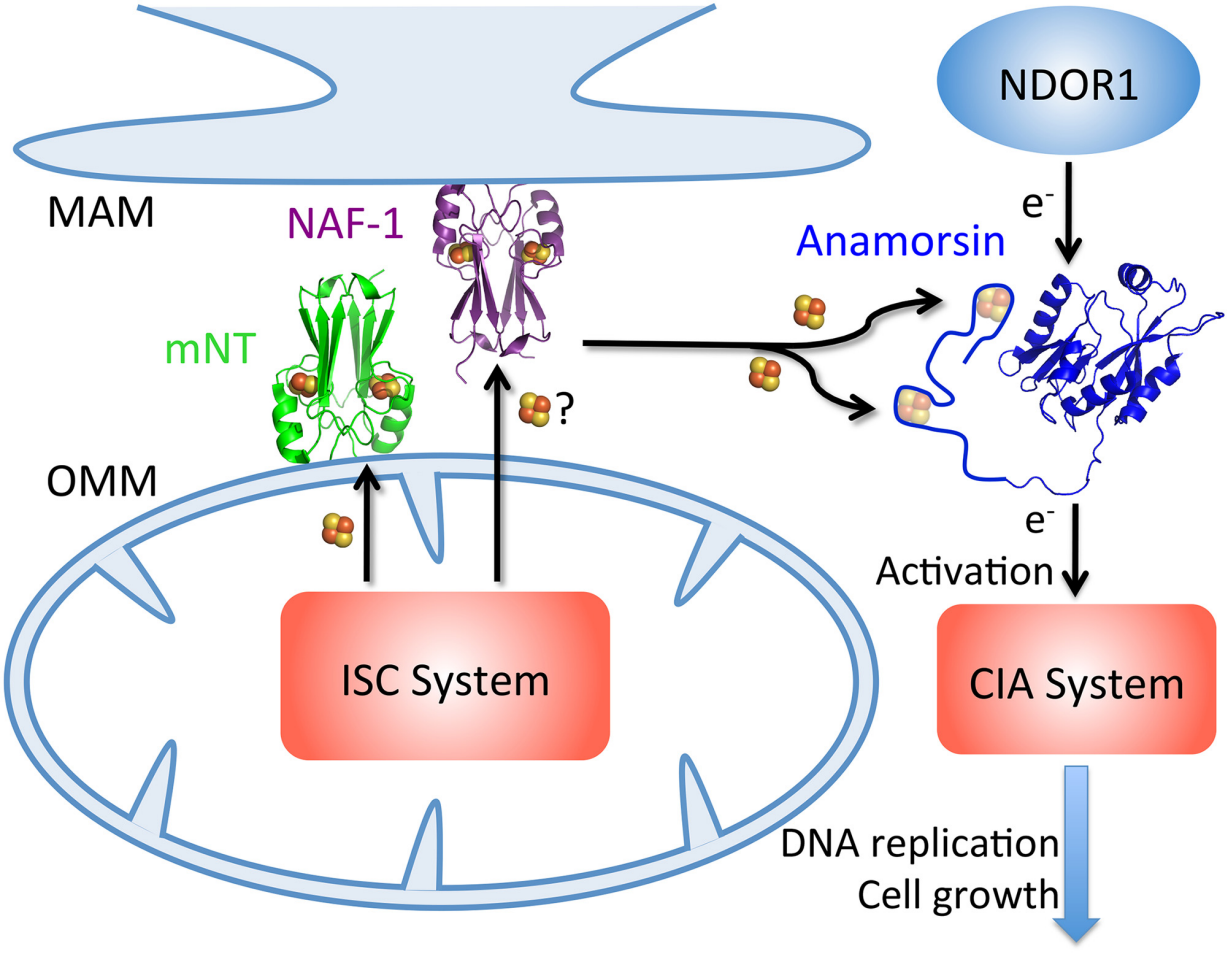
NEET proteins provide a link between the ISC and CIA pathways. mNT on the OMM and NAF-1 on the MAM receive 2Fe-2S clusters produced inside the mitochondria by the ISC system. Both mNT and NAF-1 transfer these 2Fe-2S clusters to Anamorsin. Anamorsin receives electrons from the diflavin reductase NDOR1 and supplies them to the CIA system as an early step necessary for the production of 4Fe-4S clusters. This step requires the holo form of Anamorsin. Both mNT and NAF-1 can provide parallel routes linking CIA to ISC. CIA produced clusters are targeted to proteins in the cytosol and the nucleus and are important for cell metabolism, maintenance and proliferation.

In this study, we identified Anamorsin as the first human 2Fe-2S cluster acceptor protein for both the OMM cancer related mNT and NAF-1 proteins. Recently, Ferecatu *et al*. [37] determined that mNT acquires its 2Fe-2S clusters from the mitochondrial ISC system. Our results show that once obtained, mNT (and NAF-1) can transfer its 2Fe-2S clusters to Anamorsin thereby providing a well-defined pathway from the ISC to the CIA via the outer membrane tethered mNT (Fig 7).

Anamorsin, which is an essential component of the CIA, harbors two Fe-S cluster-binding sites and we found that mNT or NAF-1 can transfer 2Fe-2S clusters to both cluster-binding sites. The NEET proteins can supply all necessary clusters for activating Anamorsin for its electron transfer function. This now links the necessity for mitochondrial Fe-S cluster assembly with the function of the cytosolic CIA [37, 38].

The NEET’s location at the OMM/MAM and their cluster transfer function similarities to ISCU suggest that they may be part of a pathway linking mitochondrial 2Fe-2S assembly to the CIA system. While Ferecatu *et al*. demonstrated that knockdown of mNT did not have a significant impact on the maturation of CIA-dependant Fe-S proteins [37], we show that NAF-1 is more efficient at transferring to apo-Anamorsin and may be a parallel route to the CIA.

The properties of the NEET proteins suggest that in addition to mitochondrial 2Fe-2S transport, they function as reservoirs allowing 2Fe-2S clusters to be assembled under favorable conditions inside the mitochondria and stored for faster availability when cytosolic 2Fe-2S clusters are needed. This would allow for faster response to immediate needs than would be possible if mitochondrial assembly and transport through two membranes to cytosolic proteins. In support of this function, mNT was recently found to reassemble/repair the 4Fe-4S cluster of cytosolic iron regulatory protein 1 (IRP1; cytosolic aconitase) following oxidative damage [37]. Thus the NEETs can function in transport of 2Fe-2S clusters out of the mitochondria as well as a reservoir of readily available 2Fe-2S clusters.

NAF-1, mNT and Anamorsin have each been implicated in cancer [15, 39–41]. Both NEET proteins are over-expressed in epithelial breast cancer cells, and decreasing their expression via shRNA knockdowns results in decreased cancer cell proliferation and decreased tumor growth. As Fe-S proteins are essential for DNA replication and cell growth, the need for fast supply of Fe-S clusters conveniently links the NEET proteins to cancer cell proliferation. Furthermore, the yeast homolog of Anamorsin (Dre2) is required for assembly of the diferric-tyrosyl radical cofactor of ribonucleotide reductase [42], which is necessary for the production of deoxynucleotides. A limited availability of deoxynucleotides would also inhibit DNA replication and thus cancer cell proliferation. Targeting the NEET proteins may be a convenient means to control many processes that are required for the accelerated proliferation of cancer cells with the possibility to modulate using small molecule targeting of the NEET proteins [43].

In this study, we provide a well-defined link between the ISC and CIA systems via mNT and NAF-1, which are the only known 2Fe-2S proteins associated with the OMM. We show that the NEET proteins can transfer both of their 2Fe-2S clusters via a direct protein-protein interaction to activate the essential CIA protein, Anamorsin.

## Supporting Information

S1 Fig UV-Vis Spectra of NEET 2Fe-2S cluster transfer to apo-Anamorsin. Spectra at select time points from the cluster transfer curves shown in Fig 2 for NAF-1 (A) and mNT (B) to apo-Anamorsin are shown. Baselines were normalized to an absorbance of zero at 800 nm.
(TIF)

S2 Fig NEET cluster transfer occurs after removal of DTT. 100 μM apo-Anamorsin was pre-incubated with 2.5 mM DTT for 60 minutes. DTT was removed using a Quick Spin Protein buffer exchange column (Roche), followed by addition of 25 μM NAF-1. Sample was kept under nitrogen to prevent disulfide bond formation. The time points shown are at 0 (red) and 200 (blue) minutes.
(TIF)

S3 Fig Comparison of oxidized and reduced NEET UV-Vis spectra. Absorbance spectrum of 25 μM NAF-1 with (red) and without (blue) 5 mM dithionite.
(TIF)

S4 Fig UV-Vis Spectra of NEET mutants. Absorption spectra of 25 μM NAF-1 H114C mutant (left) and 25 μM mNT H87C mutant. Note that the mutants show an increased absorbance at 423 nm due to the change in the ligation of the 2Fe-2S clusters, but also that the spectra are distinct from that of anamorsin (Fig 1).
(TIF)

S5 Fig NEET cluster transfer occurs in the presence of EDTA. For all traces shown 50 μM apo-Anamorsin was pre-incubated with 2.5 mM DTT 250 μM EDTA for 60 minutes followed by the addition of 100 μM ferric chloride and 100 μM ammonium sulfide (shown in red on both A and B), 25 μM NAF-1 (A) or 25 μM mNT (B). The time points shown are each at 150 minutes. No Anamorsin cluster formation occurs from free Fe^3+^ and S^2-^ in the presence of EDTA, but cluster transfer from both NAF-1 and mNT does occur.
(TIF)

S6 Fig Holo-NEETs bind to holo-Anamorsin. Biolayer interferiometry sensorgrams for the binding of 16 μM holo-NAF-1 (A) and 32 μM holo-mNT (B) to biotinylated holo-Anamorsin (prepared via cluster transfer) immobilized to streptavidin-coated biosensors are shown. The association was followed for 900 seconds (rising signal) followed by 1800 seconds of dissociation (decaying signal). The data fit best to a 2:1 heterogeneous ligand model (black curves), possibly due to a small population single cluster Anamorsin present, which can be seen in the ESI-MS spectrum in Fig 6. (C) On- and off-rates shown in the table are the average of two trials. NAF-1 binds to holo-Anamorsin with similar kinetics as to apo-Anamorsin, while mNT has a similar on-rate but a 50-fold faster off-rate. The binding between the holo-NEETs and holo-Anamorsin may have an additional biological function, possibly electron transfer, which is a subject for future studies.
(TIF)

S7 Fig UV-Vis Spectra of Anamorsin single-cluster mutants. Absorption spectra of 60 μM holo-Anamorsin-C1 mutant (A) and 90 μM holo-Anamorsin-C2 (B).
(TIF)

## References

[1] Lill R. Function and biogenesis of iron-sulphur proteins. Nature. 2009;460(7257):831–8. 10.1038/nature08301 19675643

[2] Fuss JO, Tsai CL, Ishida JP, Tainer JA. Emerging critical roles of Fe-S clusters in DNA replication and repair. Biochim Biophys Acta. 2015;1853(6):1253–71. 10.1016/j.bbamcr.2015.01.018 25655665 PMC4576882

[3] Maio N, Rouault TA. Iron-sulfur cluster biogenesis in mammalian cells: New insights into the molecular mechanisms of cluster delivery. Biochim Biophys Acta. 2015;1853(6):1493–512. 10.1016/j.bbamcr.2014.09.009 25245479 PMC4366362

[4] Paul VD, Lill R. Biogenesis of cytosolic and nuclear iron-sulfur proteins and their role in genome stability. Biochim Biophys Acta. 2015;1853(6):1528–39. 10.1016/j.bbamcr.2014.12.018 25583461

[5] Netz DJ, Mascarenhas J, Stehling O, Pierik AJ, Lill R. Maturation of cytosolic and nuclear iron-sulfur proteins. Trends Cell Biol. 2014;24(5):303–12. 10.1016/j.tcb.2013.11.005 24314740

[6] Rouault TA. Mammalian iron-sulphur proteins: novel insights into biogenesis and function. Nat Rev Mol Cell Biol. 2015;16(1):45–55. 10.1038/nrm3909 25425402

[7] Sheftel A, Stehling O, Lill R. Iron-sulfur proteins in health and disease. Trends Endocrinol Metabol. 2010;21(5):302–14. 10.1016/j.tem.2009.12.006 20060739

[8] Tamir S, Paddock ML, Darash-Yahana-Baram M, Holt SH, Sohn YS, Agranat L, et al Structure-function analysis of NEET proteins uncovers their role as key regulators of iron and ROS homeostasis in health and disease. Biochim Biophys Acta. 2015;1853(6):1294–315. 10.1016/j.bbamcr.2014.10.014 25448035

[9] Chang NC, Nguyen M, Germain M, Shore GC. Antagonism of Beclin 1-dependent autophagy by BCL-2 at the endoplasmic reticulum requires NAF-1. EMBO J. 2010;29(3):606–18. 10.1038/emboj.2009.369 20010695 PMC2830692

[10] Wiley SE, Murphy AN, Ross SA, van der Geer P, Dixon JE. MitoNEET is an iron-containing outer mitochondrial membrane protein that regulates oxidative capacity. Proc Natl Acad Sci USA. 2007;104(13):5318–23. 17376863 10.1073/pnas.0701078104 PMC1838440

[11] Wiley SE, Andreyev AY, Divakaruni AS, Karisch R, Perkins G, Wall EA, et al Wolfram Syndrome protein, Miner1, regulates sulphydryl redox status, the unfolded protein response, and Ca2+ homeostasis. EMBO Mol Med. 2013;5(6):904–18. 10.1002/emmm.201201429 23703906 PMC3779451

[12] Szabadkai G, Bianchi K, Varnai P, De Stefani D, Wieckowski MR, Cavagna D, et al Chaperone-mediated coupling of endoplasmic reticulum and mitochondrial Ca2+ channels. J Cell Biol. 2006;175(6):901–11. 17178908 10.1083/jcb.200608073 PMC2064700

[13] Chang NC, Nguyen M, Bourdon J, Risse PA, Martin J, Danialou G, et al Bcl-2-associated autophagy regulator Naf-1 required for maintenance of skeletal muscle. Hum Mol Gen. 2012;21(10):2277–87. 10.1093/hmg/dds048 22343142

[14] Colca JR, McDonald WG, Waldon DJ, Leone JW, Lull JM, Bannow CA, et al Identification of a novel mitochondrial protein ("mitoNEET") cross-linked specifically by a thiazolidinedione photoprobe. Am J Physiol Endocrinol Metab. 2004;286(2):E252–60. 14570702 10.1152/ajpendo.00424.2003

[15] Sohn YS, Tamir S, Song L, Michaeli D, Matouk I, Conlan AR, et al NAF-1 and mitoNEET are central to human breast cancer proliferation by maintaining mitochondrial homeostasis and promoting tumor growth. Proc Natl Acad Sci USA. 2013;110(36):14676–81. 10.1073/pnas.1313198110 23959881 PMC3767537

[16] Taminelli GL, Sotomayor V, Valdivieso AG, Teiber ML, Marin MC, Santa-Coloma TA. CISD1 codifies a mitochondrial protein upregulated by the CFTR channel. Biochem Biophysical Res Commun. 2008;365(4):856–62. 10.1016/j.bbrc.2007.11.076 18047834

[17] Chen YF, Kao CH, Chen YT, Wang CH, Wu CY, Tsai CY, et al Cisd2 deficiency drives premature aging and causes mitochondria-mediated defects in mice. Genes Dev. 2009;23(10):1183–94. 10.1101/gad.1779509 19451219 PMC2685531

[18] Amr S, Heisey C, Zhang M, Xia XJ, Shows KH, Ajlouni K, et al A homozygous mutation in a novel zinc-finger protein, ERIS, is responsible for Wolfram syndrome 2. Am J Hum Genet. 2007;81(4):673–83. 17846994 10.1086/520961 PMC2227919

[19] Conlan AR, Axelrod HL, Cohen AE, Abresch EC, Zuris J, Yee D, et al Crystal structure of Miner1: The redox-active 2Fe-2S protein causative in Wolfram Syndrome 2. J Mol Biol. 2009;392(1):143–53. 10.1016/j.jmb.2009.06.079 19580816 PMC2739586

[20] Paddock ML, Wiley SE, Axelrod HL, Cohen AE, Roy M, Abresch EC, et al MitoNEET is a uniquely folded 2Fe 2S outer mitochondrial membrane protein stabilized by pioglitazone. Proc Natl Acad Sci USA. 2007;104(36):14342–7. 17766440 10.1073/pnas.0707189104 PMC1963346

[21] Qi W, Cowan JA. Structural, Mechanistic and Coordination Chemistry of Relevance to the Biosynthesis of Iron-Sulfur and Related Iron Cofactors. Coord Chem Rev. 2011;255(7–8):688–99. 21499539 10.1016/j.ccr.2010.10.016 PMC3074115

[22] Tamir S, Zuris JA, Agranat L, Lipper CH, Conlan AR, Michaeli D, et al Nutrient-deprivation autophagy factor-1 (NAF-1): biochemical properties of a novel cellular target for anti-diabetic drugs. PLoS ONE. 2013;8(5):e61202 10.1371/journal.pone.0061202 23717386 PMC3661554

[23] Zuris JA, Harir Y, Conlan AR, Shvartsman M, Michaeli D, Tamir S, et al Facile transfer of [2Fe-2S] clusters from the diabetes drug target mitoNEET to an apo-acceptor protein. Proc Natl Acad Sci USA. 2011;108(32):13047–52. 10.1073/pnas.1109986108 21788481 PMC3156153

[24] Banci L, Bertini I, Calderone V, Ciofi-Baffoni S, Giachetti A, Jaiswal D, et al Molecular view of an electron transfer process essential for iron-sulfur protein biogenesis. Proc Natl Acad Sci USA. 2013;110(18):7136–41. 10.1073/pnas.1302378110 23596212 PMC3645582

[25] Netz DJ, Stumpfig M, Dore C, Muhlenhoff U, Pierik AJ, Lill R. Tah18 transfers electrons to Dre2 in cytosolic iron-sulfur protein biogenesis. Nat Chem biol. 2010;6(10):758–65. 10.1038/nchembio.432 20802492

[26] Zhang Y, Lyver ER, Nakamaru-Ogiso E, Yoon H, Amutha B, Lee DW, et al Dre2, a conserved eukaryotic Fe/S cluster protein, functions in cytosolic Fe/S protein biogenesis. Mol Cell Biol. 2008;28(18):5569–82. 10.1128/MCB.00642-08 18625724 PMC2546940

[27] Banci L, Bertini I, Ciofi-Baffoni S, Boscaro F, Chatzi A, Mikolajczyk M, et al Anamorsin is a [2Fe-2S] cluster-containing substrate of the Mia40-dependent mitochondrial protein trapping machinery. Chem Biol. 2011;18(6):794–804. 10.1016/j.chembiol.2011.03.015 21700214

[28] Kennedy MC, Beinert H. The state of cluster SH and S2- of aconitase during cluster interconversions and removal. A convenient preparation of apoenzyme. J Biol Chem. 1988;263(17):8194–8. 2836417

[29] Nechushtai R, Conlan AR, Harir Y, Song L, Yogev O, Eisenberg-Domovich Y, et al Characterization of Arabidopsis NEET reveals an ancient role for NEET proteins in iron metabolism. Plant Cell. 2012;24(5):2139–54. 10.1105/tpc.112.097634 22562611 PMC3442592

[30] Landry AP, Ding H. Redox Control of Human Mitochondrial Outer Membrane Protein MitoNEET [2Fe-2S] Clusters by Biological Thiols and Hydrogen Peroxide. J Biol Chem. 2014;289(7):4307–15. 10.1074/jbc.M113.542050 24403080 PMC3924293

[31] Vranish JN, Russell WK, Yu LE, Cox RM, Russell DH, Barondeau DP. Fluorescent probes for tracking the transfer of iron-sulfur cluster and other metal cofactors in biosynthetic reaction pathways. J Am Chem Soc. 2015;137(1):390–8. 10.1021/ja510998s 25478817 PMC4675328

[32] Wu SP, Wu G, Surerus KK, Cowan JA. Iron-sulfur cluster biosynthesis. Kinetic analysis of [2Fe-2S] cluster transfer from holo ISU to apo Fd: role of redox chemistry and a conserved aspartate. Biochemistry. 2002;41(28):8876–85. 12102630 10.1021/bi0256781

[33] Tamir S, Eisenberg-Domovich Y, Conlan AR, Stofleth JT, Lipper CH, Paddock ML, et al A point mutation in the [2Fe-2S] cluster binding region of the NAF-1 protein (H114C) dramatically hinders the cluster donor properties. Acta crystallogr Sect D Biol Cryst. 2014;70(Pt 6):1572–8. 24914968 10.1107/S1399004714005458 PMC4051502

[34] Conlan AR, Paddock ML, Homer C, Axelrod HL, Cohen AE, Abresch EC, et al Mutation of the His ligand in mitoNEET stabilizes the 2Fe-2S cluster despite conformational heterogeneity in the ligand environment. Acta crystallogr Sect D Biol Cryst. 2011;67(Pt 6):516–23. 21636891 10.1107/S0907444911011577 PMC3107049

[35] Wu SP, Cowan JA. Iron-sulfur cluster biosynthesis. A comparative kinetic analysis of native and Cys-substituted ISA-mediated [2Fe-2S]^2+^ cluster transfer to an apoferredoxin target. Biochemistry. 2003;42(19):5784–91. 12741836 10.1021/bi026939+

[36] Chandramouli K, Johnson MK. HscA and HscB stimulate [2Fe-2S] cluster transfer from IscU to apoferredoxin in an ATP-dependent reaction. Biochemistry. 2006;45(37):11087–95. 16964969 10.1021/bi061237w PMC2518968

[37] Ferecatu I, Goncalves S, Golinelli-Cohen MP, Clemancey M, Martelli A, Riquier S, et al The Diabetes Drug Target MitoNEET Governs a Novel Trafficking Pathway to Rebuild an Fe-S Cluster into Cytosolic Aconitase/Iron Regulatory Protein 1. J Biol Chem. 2014;289(41):28070–86. 10.1074/jbc.M114.548438 25012650 PMC4192461

[38] Lill R, Srinivasan V, Muhlenhoff U. The role of mitochondria in cytosolic-nuclear iron-sulfur protein biogenesis and in cellular iron regulation. Curr Op Microbiol. 2014;22C:111–9. 10.1016/j.mib.2014.09.015 25460804

[39] Lu D, Xiao Z, Wang W, Xu Y, Gao S, Deng L, et al Down regulation of CIAPIN1 reverses multidrug resistance in human breast cancer cells by inhibiting MDR1. Molecules. 2012;17(6):7595–611. 10.3390/molecules17067595 22717413 PMC6268881

[40] Li X, Pan Y, Fan R, Jin H, Han S, Liu J, et al Adenovirus-delivered CIAPIN1 small interfering RNA inhibits HCC growth in vitro and in vivo. Carcinogenesis. 2008;29(8):1587–93. 10.1093/carcin/bgn052 18299278 PMC2516489

[41] Liu L, Xia M, Wang J, Zhang W, Zhang Y, He M. CISD2 expression is a novel marker correlating with pelvic lymph node metastasis and prognosis in patients with early-stage cervical cancer. Med Oncol. 2014;31(9):183 10.1007/s12032-014-0183-5 25134919

[42] Zhang Y, Li H, Zhang C, An X, Liu L, Stubbe J, et al Conserved electron donor complex Dre2-Tah18 is required for ribonucleotide reductase metallocofactor assembly and DNA synthesis. Proc Natl Acad Sci USA. 2014;111(17):E1695–704. 10.1073/pnas.1405204111 24733891 PMC4035922

[43] Bai F, Morcos F, Sohn YS, Darash-Yahana M, Rezende CO, Lipper CH, et al The Fe-S cluster-containing NEET proteins mitoNEET and NAF-1 as chemotherapeutic targets in breast cancer. Proc Natl Acad Sci USA. 2015;112(12):3698–703. 10.1073/pnas.1502960112 25762074 PMC4378444

