# Supplementary figures and images for: Cancer-Related NEET Proteins Transfer 2Fe-2S Clusters to Anamorsin, a Protein Required for Cytosolic Iron-Sulfur Cluster Biogenesis
Source: PLoS One. 2015 Oct 8;10(10):e0139699. doi: 10.1371/journal.pone.0139699 (PMC4598119; doi:10.1371/journal.pone.0139699)

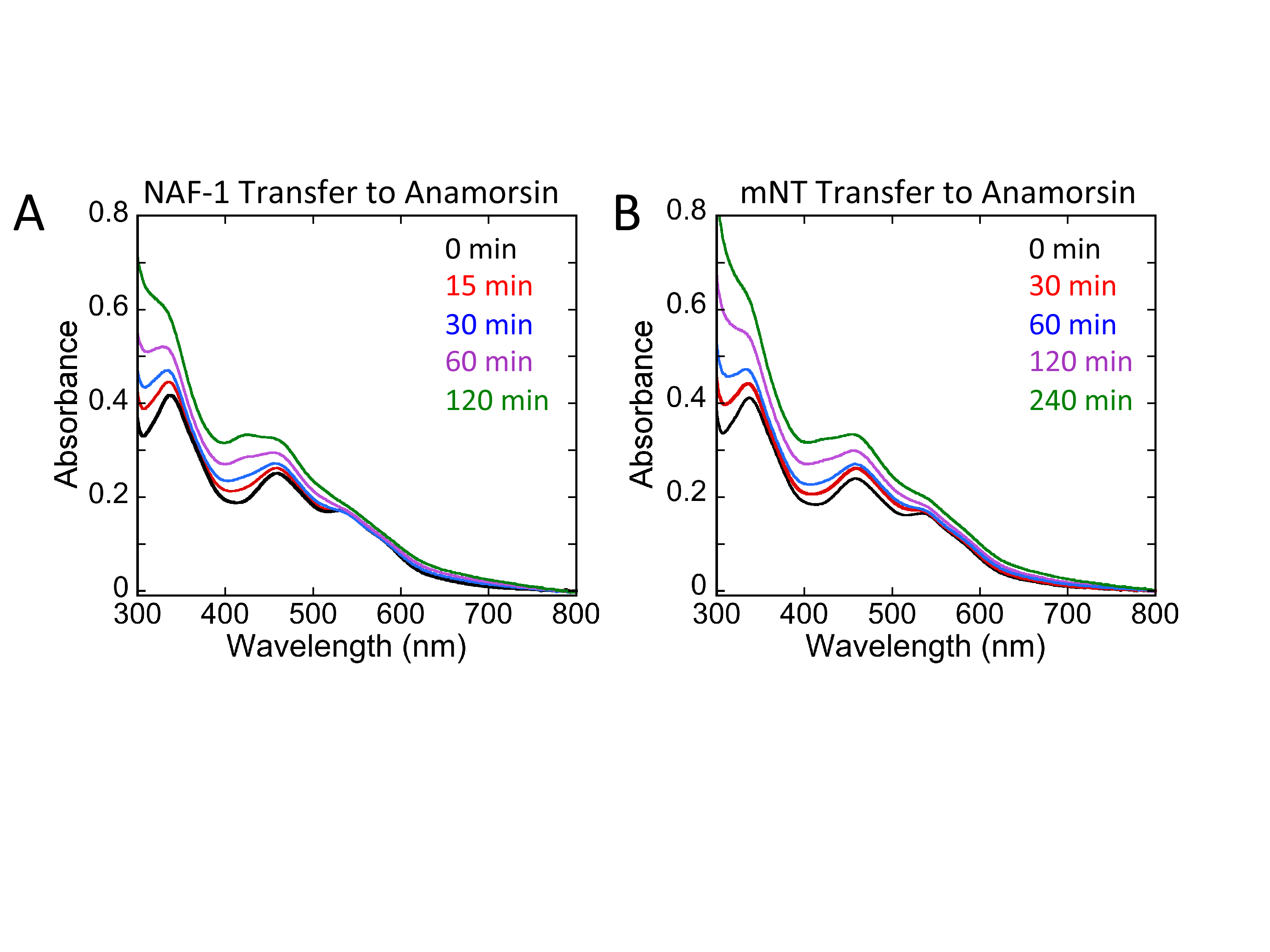

Supplement: S1 Fig — Spectra at select time points from the cluster transfer curves shown in Fig 2 for NAF-1 (A) and mNT (B) to apo-Anamorsin are shown. Baselines were normalized to an absorbance of zero at 800 nm. (TIF) [file pone.0139699.s001.tif]

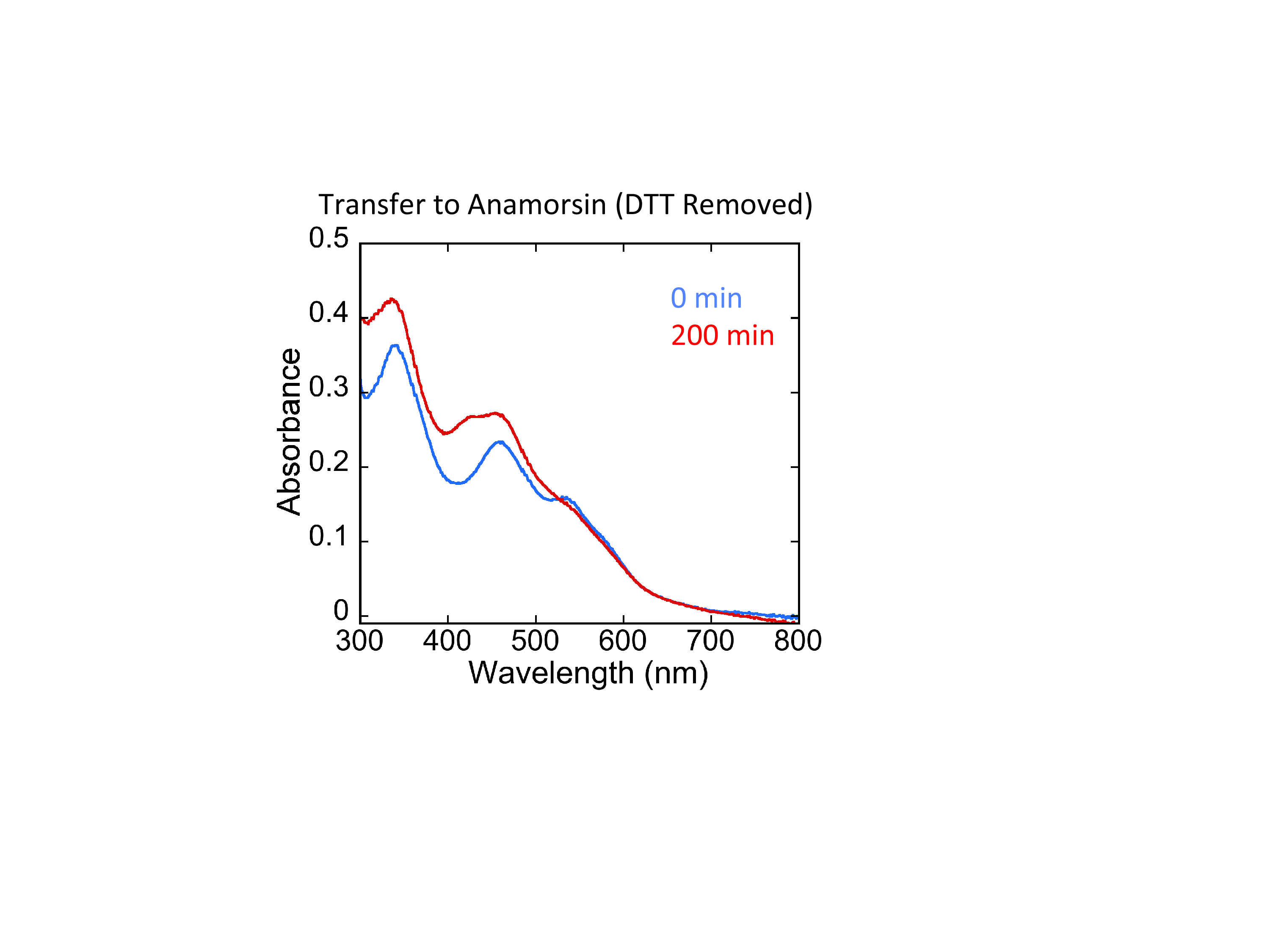

Supplement: S2 Fig — 100 μM apo-Anamorsin was pre-incubated with 2.5 mM DTT for 60 minutes. DTT was removed using a Quick Spin Protein buffer exchange column (Roche), followed by addition of 25 μM NAF-1. Sample was kept under nitrogen to prevent disulfide bond formation. The time points shown are at 0 (red) and 200 (blue) minutes. (TIF) [file pone.0139699.s002.tif]

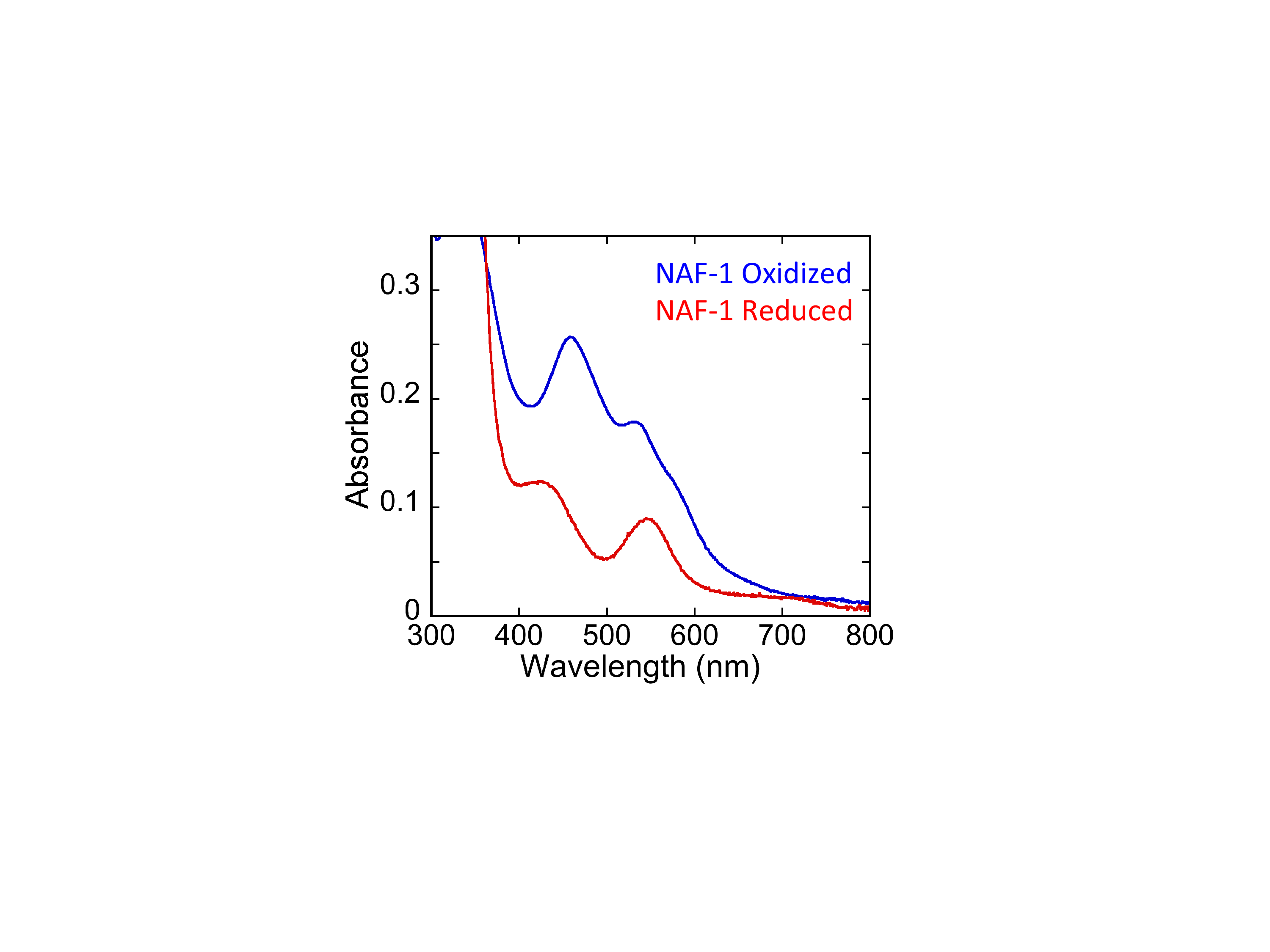

Supplement: S3 Fig — Absorbance spectrum of 25 μM NAF-1 with (red) and without (blue) 5 mM dithionite. (TIF) [file pone.0139699.s003.tif]

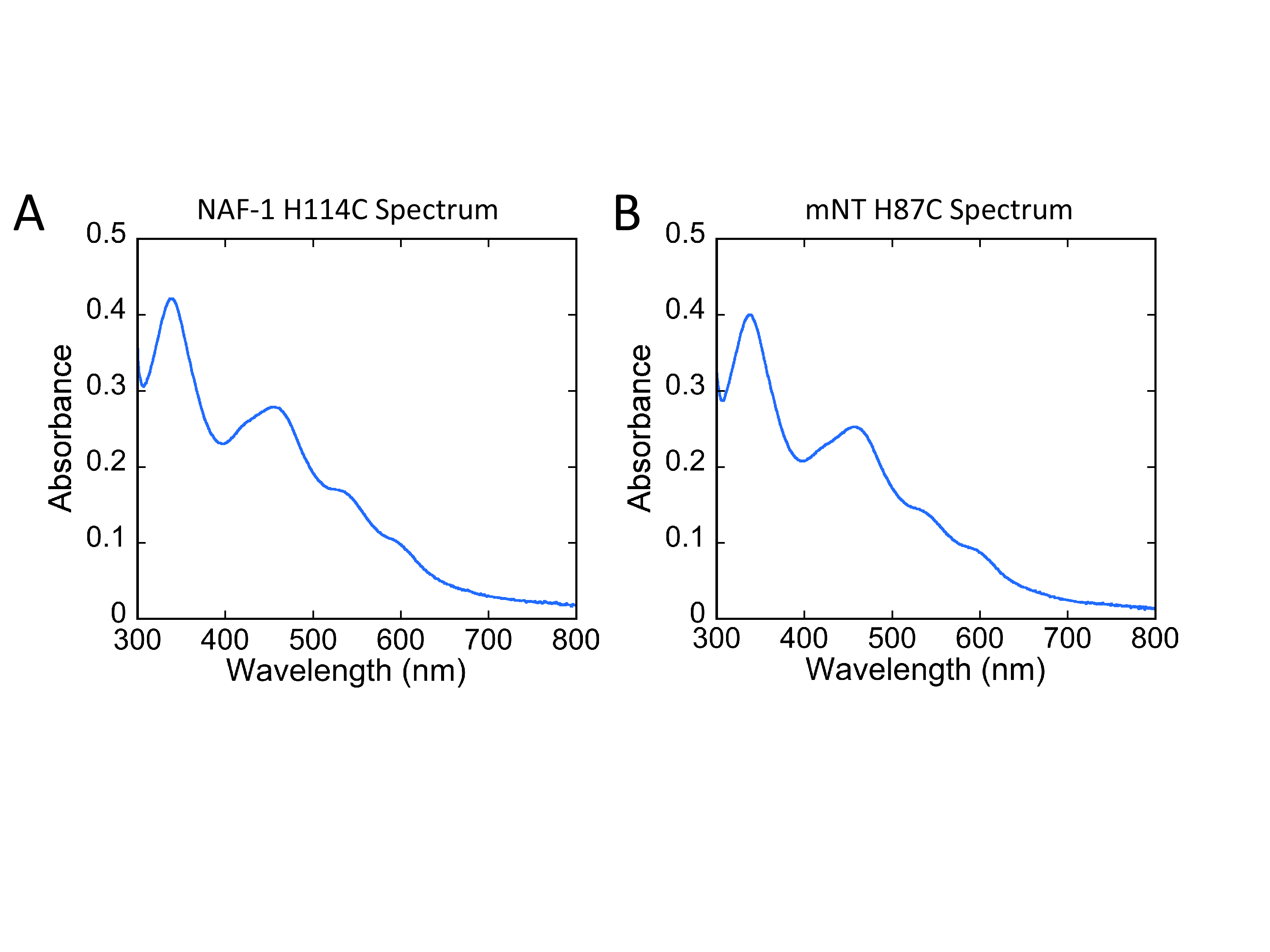

Supplement: S4 Fig — Absorption spectra of 25 μM NAF-1 H114C mutant (left) and 25 μM mNT H87C mutant. Note that the mutants show an increased absorbance at 423 nm due to the change in the ligation of the 2Fe-2S clusters, but also that the spectra are distinct from that of anamorsin (Fig 1). (TIF) [file pone.0139699.s004.tif]

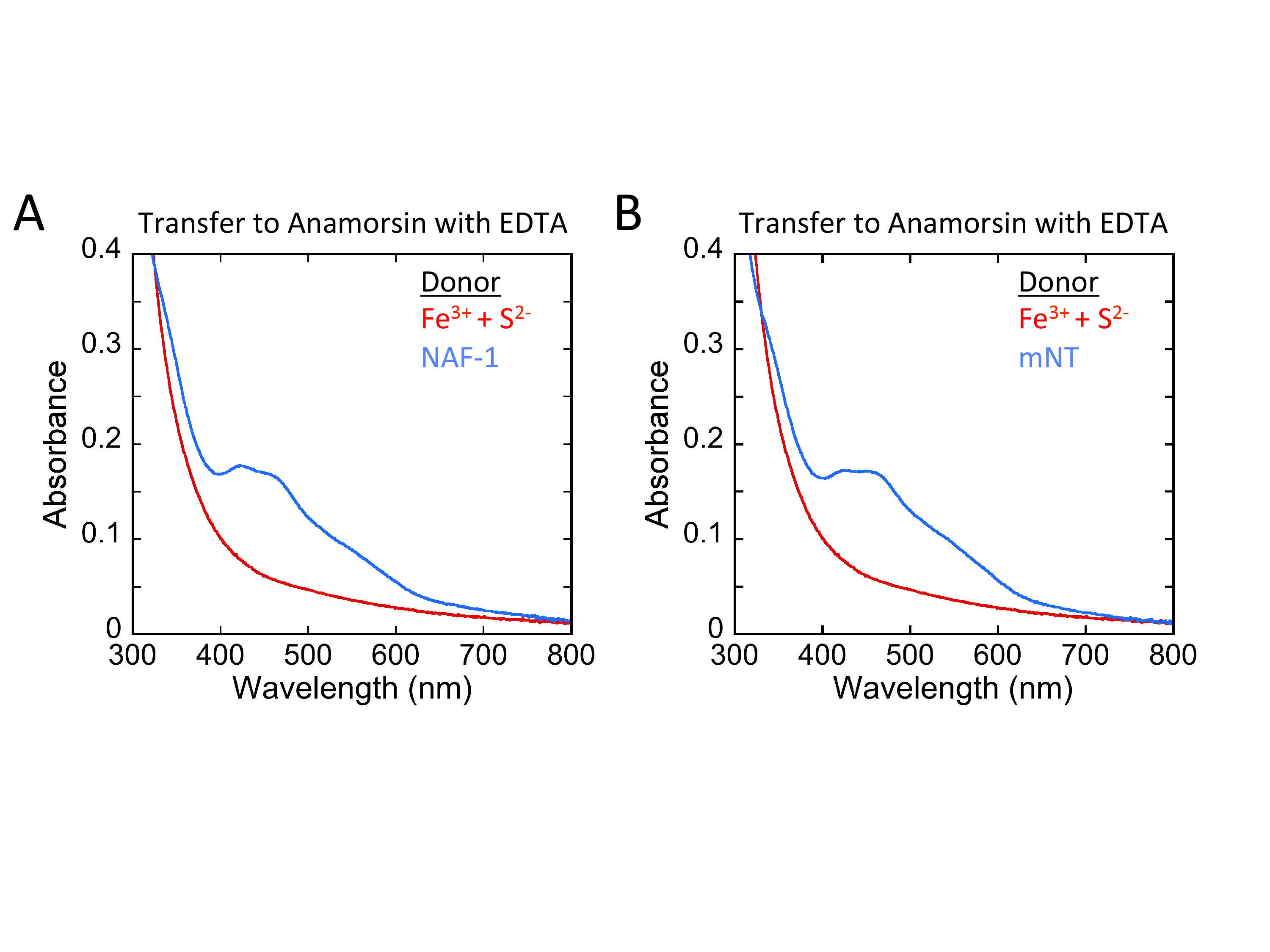

Supplement: S5 Fig — For all traces shown 50 μM apo-Anamorsin was pre-incubated with 2.5 mM DTT 250 μM EDTA for 60 minutes followed by the addition of 100 μM ferric chloride and 100 μM ammonium sulfide (shown in red on both A and B), 25 μM NAF-1 (A) or 25 μM mNT (B). The time points shown are each at 150 minutes. No Anamorsin cluster formation occurs from free Fe3+ and S2- in the presence of EDTA, but cluster transfer from both NAF-1 and mNT does occur. (TIF) [file pone.0139699.s005.tif]

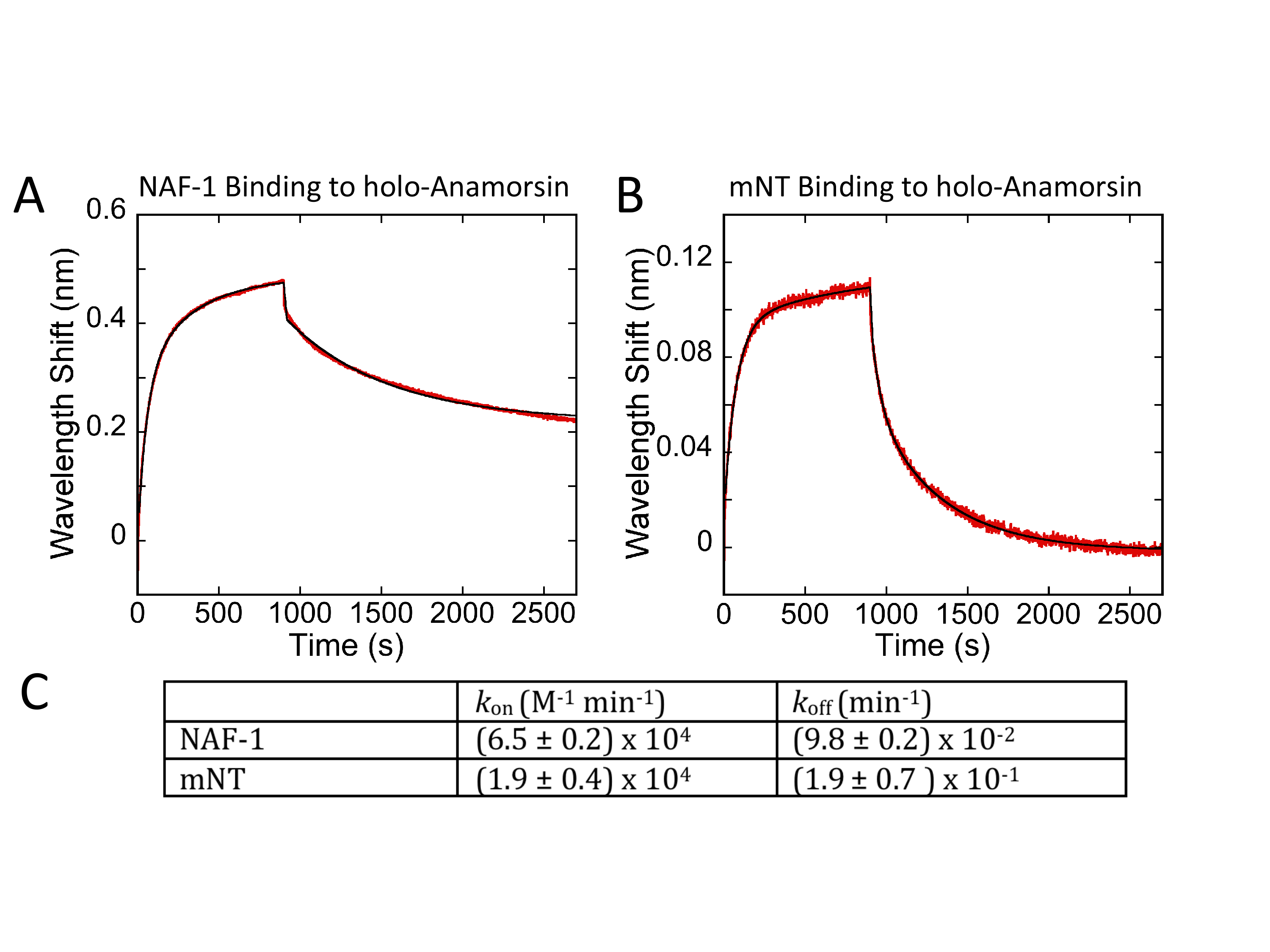

Supplement: S6 Fig — Biolayer interferiometry sensorgrams for the binding of 16 μM holo-NAF-1 (A) and 32 μM holo-mNT (B) to biotinylated holo-Anamorsin (prepared via cluster transfer) immobilized to streptavidin-coated biosensors are shown. The association was followed for 900 seconds (rising signal) followed by 1800 seconds of dissociation (decaying signal). The data fit best to a 2:1 heterogeneous ligand model (black curves), possibly due to a small population single cluster Anamorsin present, which can be seen in the ESI-MS spectrum in Fig 6. (C) On- and off-rates shown in the table are the average of two trials. NAF-1 binds to holo-Anamorsin with similar kinetics as to apo-Anamorsin, while mNT has a similar on-rate but a 50-fold faster off-rate. The binding between the holo-NEETs and holo-Anamorsin may have an additional biological function, possibly electron transfer, which is a subject for future studies. (TIF) [file pone.0139699.s006.tif]

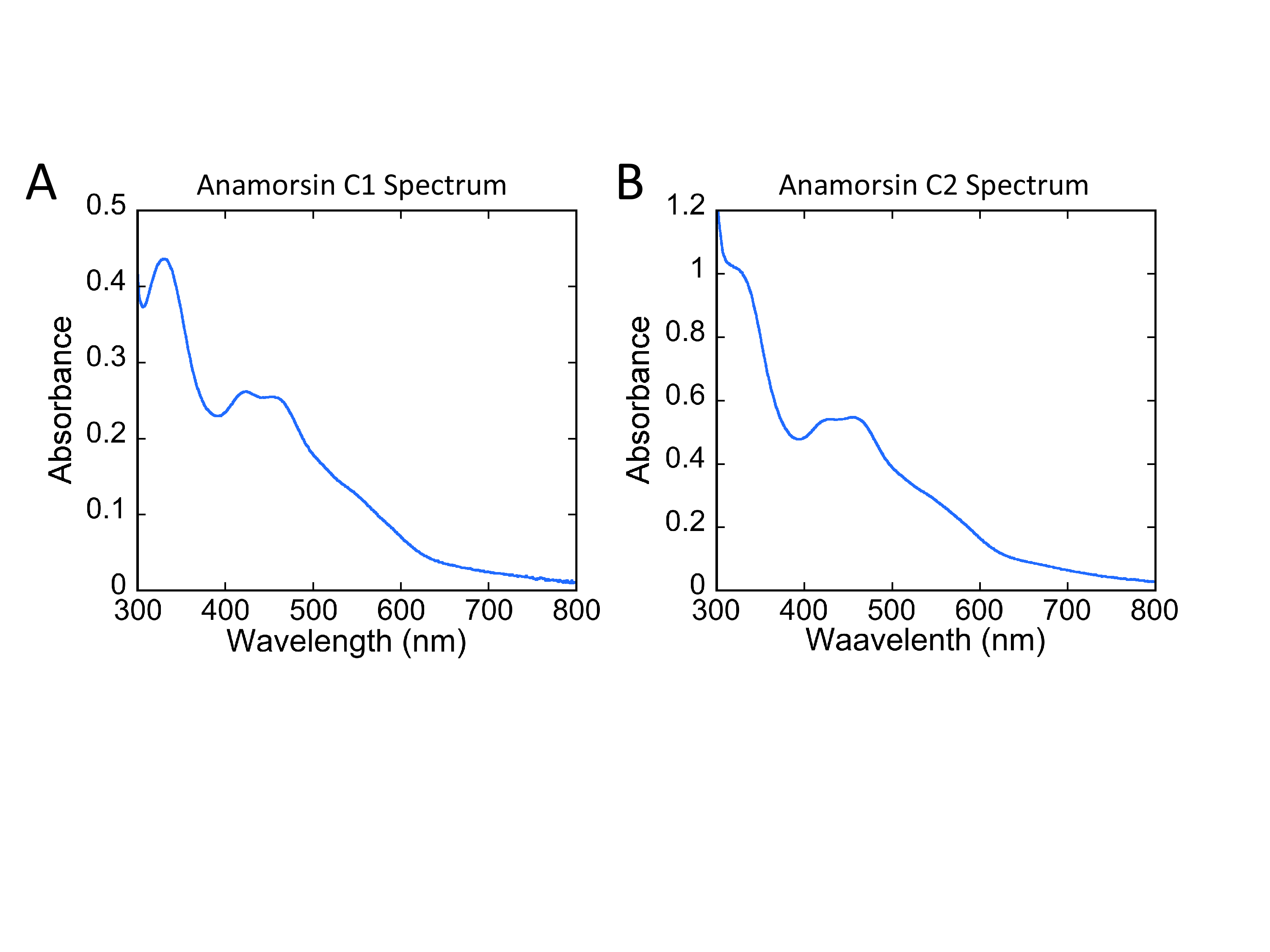

Supplement: S7 Fig — Absorption spectra of 60 μM holo-Anamorsin-C1 mutant (A) and 90 μM holo-Anamorsin-C2 (B). (TIF) [file pone.0139699.s007.tif]
